# Lanthanum exposure and its metabolomic effects on *Ruditapes philippinarum*

**DOI:** 10.1038/s41598-025-15576-7

**Published:** 2025-09-26

**Authors:** Fabrizio Mastrorocco, Marco Vito Guglielmi, Luca De Martino, Clara Musicco, Sharon Natasha Cox, Graziano Pesole, Giovanni Scillitani, Claudia Leoni

**Affiliations:** 1 Institute of Biomembranes, Bioenergetics and Molecular Biotechnologies (IBIOM), National Research Council (CNR), Via Amendola n. 122/O, Bari, 70126 Italy; 2 Water Research Institute (IRSA) National Research Council (CNR), Taranto, Italy; 3 Department of Biosciences, Biotechnologies and Environment, University of Bari A. Moro, Via Orabona n. 4, Bari, 70126 Italy

**Keywords:** Clam, Lanthanum, Metabolites, Biomarkers, Mediterranean aquaculture, Biochemistry, Ecology, Molecular biology, Ecology, Environmental sciences

## Abstract

The growing release of rare earth elements (REEs) into aquatic environments, driven by their extensive industrial and technological applications, has raised growing concern due to their largely unknown ecological and toxicological effects. Among these, lanthanum (La), one of the most widely used REEs, is increasingly detected in marine ecosystems. This study investigates the short-term impact of exposure on the Manila clam, *Ruditapes philippinarum*, a key benthic species in Mediterranean aquaculture. Clams were exposed to 10 mg/L of LaCl₃·7 H₂O, and their gill tissues were analyzed through histological examination and untargeted metabolomics. Histological analysis revealed epithelial degeneration, decreased mucus secretion, and altered glycoprotein expression following exposure. Metabolomic profiling identified 188 differentially expressed metabolites (DEMs) associated with disruptions in organic acid, fatty acid, amino acid, and nucleotide metabolism. Functional analysis of DEMs revealed that alterations in glutathione (GSH), ascorbic acid, oleic acid, and thymidine levels may serve as biomarkers of La-induced oxidative and metabolic stress in bivalves. These findings emphasize the ecological threats associated with the increasing REEs contamination in marine environments.

**Supplementary Information:**

The online version contains supplementary material available at 10.1038/s41598-025-15576-7.

## Introduction

The rapid globalization of production cycles and unsustainable urbanization raise the emergency for the contamination of aquatic environments with a significant impact on human health, environmental biodiversity, and climate conditions^1,2^. As a result, direct and indirect effects leading to new human, animal, and plant pathologies are observed. The most critical contaminants in aquatic environments are inorganic compounds such as metals, due to their persistence, bioaccumulation capacity, and toxicity^3^. The presence of metals is mainly caused by mining activities and due to the release of chemicals from pharmaceuticals, nanomaterials, microplastics, pesticides, flame retardants, perfluorinated compounds, and cosmetics. Most wastewater treatment plants fail to reduce the concentrations of these substances, especially in marine environments. Furthermore, monitoring chemical contaminants released into aquatic ecosystems is both expensive and challenging because they form mixtures whose risk is difficult to quantify^4^.

Among emerging contaminants, rare earth elements (REEs) are increasingly used in high-tech, medical, and agricultural processes. This extensive use leads to significant releases of their compounds into the environment, particularly in aquatic ecosystems worldwide, with concentrations often exceeding permitted levels^5,6^. Compared to other metals^7^, knowledge of the toxicity of REEs in marine organisms remains limited. In particular, a significant increase in lanthanum (La) concentrations has recently been recorded in aquatic environments due to its extensive use in various industries, including glass production, rechargeable batteries, fluorescent lights, and other applications requiring colored light^8–10^. La is a REEs element belonging to the lanthanide group, characterized by low solubility and a tendency to precipitate or bind to complexing ions with low solubility^11^. Several studies have also detected the presence of La in marine organisms, including amphipods, bivalves, and snails^12–15^. When present at high concentrations in aquatic environments, La can have toxic effects on marine life. It can cause cellular damage to the mitochondria and nucleus, potentially leading to the organism death or altering the function of some enzymes^16–19^. Indeed, high concentrations of La have a detrimental impact on the Mediterranean mussel, *Mytilus galloprovincialis*, affecting not only its biochemical performance but also its reproductive capacity and growth rate. This exposure leads to oxidative and neurotoxic stress in the organism^19^. Currently, there is no available information regarding the impact of La on clams. In many bivalves including members of the Veneridae family, the gills extend along the full length of the animal and perform multiple essential functions. They are not only responsible for respiration and osmoregulation but also play a key role in feeding by transporting food particles from the siphonal region toward the mouth and eliminating non-digestible material via pseudofeces. Importantly, due to their large surface area and continuous direct exposure to the surrounding water, gills are particularly susceptible to environmental contaminants, making them a sensitive and reliable tissue for detecting toxicological effects. For this reason, gills represent a strategic target for evaluating the physiological and biochemical impact of pollutant exposure in bivalves. The aim of this study was to evaluate the effects of high La concentrations on the Manila clam, *R. philippinarum*, after a 3-day exposure. This bivalve, a benthic filter-feeding mollusk, is widely distributed across the globe due to its commercial value and importance in aquaculture, particularly in many regions of the Mediterranean Sea^20,21^. To investigate the effects of La treatment, we focused on the gills of *R. philippinarum* by examining histopathological changes and conducting an untargeted metabolomic analysis to assess underlying biochemical changes.

## Materials and methods

### Experimental design

Manila clams *R. philippinarum* (Bivalvia: Veneridae) were obtained commercially from a fishmonger in Bari (Italy). The clams had been caught three days earlier in Chioggia (Venice, Italy), as reported in the product traceability sheet. Acclimatization in the laboratory was performed in 105-liter tanks with 30 L artificial sea water at 15 °C for one day and fed with microalgae *Tetraselmis suecica*, as reported in previously studies^7,22^. Samples of about the same size (mean valve length 34.28 ± 2.66 mm, mean animal wet weight 11.69 ± 2.70 mg) were selected for experimentation. Two experimental groups of 20 animals each were obtained and treated using Cl_3_La·7H_2_O (Merck Life Science, Rahway, NJ, USA) at a La concentration of 0 mg/L, control samples (CTRL), and 10.0 mg/L, treated samples (La10). The reported concentrations are obtained from data present in the literature^22^. The treatments lasted for three days, during which water quality and experimental conditions were monitored. No water changes were made during this period, and the animals were fed regularly. At the conclusion of the study, only two organisms died in the La10 tank. For histochemical analysis, the gills of five samples from each group were used, while for the metabolomic study, 15 gills from the CTRL group and 13 from the La10 group were selected. Continuous variables were expressed as mean ± standard deviation (SD). Differences between the two groups were assessed using the two-tailed Student’s t-test for normally distributed data and the Mann–Whitney U test for non-normally distributed data. A p-value < 0.05 was considered statistically significant. All statistical analyses were performed using GraphPad Prism (version 8.0.2).

Animal treatments followed the European Directive 2010/63/EU on animal use for research and the Italian laws, even if bivalve mollusks like *R.philippinarum* are not mentioned in the cited Directive.

### Conventional histochemistry

Gills were fixed in 4% formalin for one hour and dehydrated through a graded ethanol series^23^. Tissue samples were embedded in paraffin wax and serially cut at 5 μm, then they were rehydrated before staining. Histochemical analyses were performed as described for the snail *Eobania vermiculata*^24^. Stains included periodic acid-Schiff (PAS), Alcian Blue pH 2.5 (AB), and high iron diamine (HID), detecting neutral, acidic, and sulfated carbohydrates, respectively. Hemalum was used to counterstain nuclei. Protocol details are available in Guglielmi et al.^24^, and all chemicals were sourced from Sigma-Aldrich Inc. (St. Louis, MO, USA).

### Lectin histochemistry

Lectin histochemistry was performed with eight FITC-conjugated lectins, - PNA, SBA, WGA, SNA, ConA, AAA, UEA-I, and LTA (Vector Laboratories, Newark, CA, United States) to characterize glycosidic chains in mucin-secreting cells (Table S1 in Supplementary Materials)^25,26^. Lectins were selected based on data about mollusks, as available in literature^27–29^. Rehydrated sections were incubated with lectins for one hour at room temperature in HEPES buffer (Sigma-Aldrich, St. Louis, MO, United States). Lectin specificity was ensured by pre-incubation of controls with inhibitory sugars, buffer-only incubations, and tissues from other species that resulted positive from previous experiments^24,25,29^. Fluorescence microscopy was used to analyze lectin-binding patterns. Bright-field histochemical stains (PAS, AB, HID) and fluorescence images from lectin-binding assays were captured using an Eclipse N*i* photomicroscope equipped with a DS-F*i*3 digital camera (Nikon Instruments SpA, Calenzano, Italy).

### Metabolite extraction

The LC-MS grade solvents utilized in this study were as follows: LC-MS Chromasolv™ Acetonitrile (ACN) (Riedel-de-Haën™), LC-MS Chromasolv™ Methanol (MeOH) (Riedel-de-Haën™), LiChropur™ Ammonium formate (NH_4_HCO_2_) (SIGMA-ALDRICH) and Optima Formic Acid (FAc) (Fisher Chemical). Internal standards were 3-Acetylindole (SIGMA-ALDRICH) and N-Acetyl-L-phenylalanine (SIGMA-ALDRICH). Dried metabolite extract of each sample was obtained from gills of CTRL and La10 clams, based on the protocol described by Yuan et al.^30^ with minor modifications. Briefly, gills clams were frozen in liquid nitrogen and homogenized with a pestle in an agate mortar. A volume of 1.5 mL of the extraction solution (MeOH/ACN/H_2_O 6:2:1, v: v:v), cooled to −80 °C, was used to recover and solubilize the samples. The solution was transferred to a 1.5 mL Eppendorf and centrifuged at 4 °C for 10 min at 13,000 RCF; the supernatant was dried in SpeedVac. The dried pellet samples were dissolved in organic solution ACN/MeOH/H_2_O (7:1:2, v: v:v) adding Internal Standards (3-Acetylindole and N-Acetyl-L-phenylalanine) as quality control. Resuspended samples were centrifuged at 4 °C for 10 min at 15,000 RCF before mass spectrometry analysis. The quality control (QC) was prepared by mixing equal volumes of each individual sample.

### Liquid chromatography/mass spectrometry (LC/MS)

Gills metabolites were analyzed using a Vanquish™ Flex UHPLC system (Thermo Scientific, Waltham, USA) coupled to an Orbitrap Fusion™ Tribrid™ mass spectrometer (Thermo Fisher Scientific) equipped with a Heated Electrospray Ion (HESI) source, as reported by Nocito et al.^31^. Samples were analyzed in a randomized manner, using a blank sample for background noise correction and pooled QC sample for data normalization. Metabolites were separated on a Thermo Fisher Accucore™ −150-Amide-HILIC column (100 × 2.1 mm; 2.6 μm). Mass spectrometry analyses were performed in positive and negative ion mode with a scan range of m/z 50 to 1,000 at a mass resolution of 120,000 for full-scan MS. Fragmentation spectra were acquired in Data-Dependent Acquisition (DDA) mode at 60,000 mass resolution via high-energy collision dissociation (HCD) for MS2 and collision-induced dissociation (CID) for MS3. To capture high - quality fragmentation spectra, we used AcquireX Deep Scan data acquisition workflow from Xcalibur™ 4.2 software (Thermo Fisher Scientific, Bremen, Germany). This workflow provided exclusion lists for blank and noise signal subtraction and inclusion lists for DDA of fragmentation spectra.

### Bioinformatics and statistical analyses

Raw data obtained by LC/MS analysis were processed using Compound Discoverer™ (CD, v. 3.3.0.5, Thermo Fisher Scientific, Bremen, Germany), following a workflow for untargeted metabolomics analysis. The parameters used in the analysis workflow were set as in Nocito et al.^31^. Compounds were annotated by comparing retention time and MSn spectra against our internal mass list database using the mzVault node in the data processing software, along with the Fragment Ion Search (FISh) scoring tool. Several cloud-based databases were queried, including Human Metabolome Database^32^, BioCyc^33^, ChEBI^34^, ChEMBL^35^, LipidMAPS^36^. The putative identification of annotated compounds corresponded to level B1 or B2^37^. All obtained metabolite values were normalized for the weight of each clam gill sample. To identify Differentially Expressed Metabolites (DEMs) between experimental groups. The criteria for differential expressions were set as a p-value < 0.05 and a Log2 Fold Change (Log2FC) > 1 or < −1. To explore overall metabolic differences between the groups, Principal Component Analysis (PCA) was performed using PAST 4.03 software. Pathway enrichment analysis was performed using the “Metabolika” tool integrated within CD.

To examine the biological significance of DEMs exposed to La10 we performed a canonical pathway analysis using Ingenuity Pathway Analysis (IPA, Ingenuity Systems, Redwood City, CA). DEMs were uploaded into the IPA tool along with their Log2FC. The tool calculates the significance of the pathways using the right-tailed Fisher’s Exact Test and then significance measures the likelihood that an association between a set of metabolites and the related pathway is not due to chance alone. To account for multiple canonical pathways tested by IPA, Fisher’s Exact Test was applied, and the pathways with a –log (correction p-value) > 2 were reported. The overall activation/inhibition states of Canonical Pathways are predicted based on a z-score algorithm which allowed us to infer the activation states of the pathways by considering the activation state of one or more key molecules within the Pathway and the molecules’ causal relationships with each other to generate an activity pattern for the molecules and the end-point functions in the pathway. We considered p-values < 0.05 and Z-scores > 1 (activation) or < −1 (inhibition).

## Results

### Histochemistry analysis

**Fig. 1:**
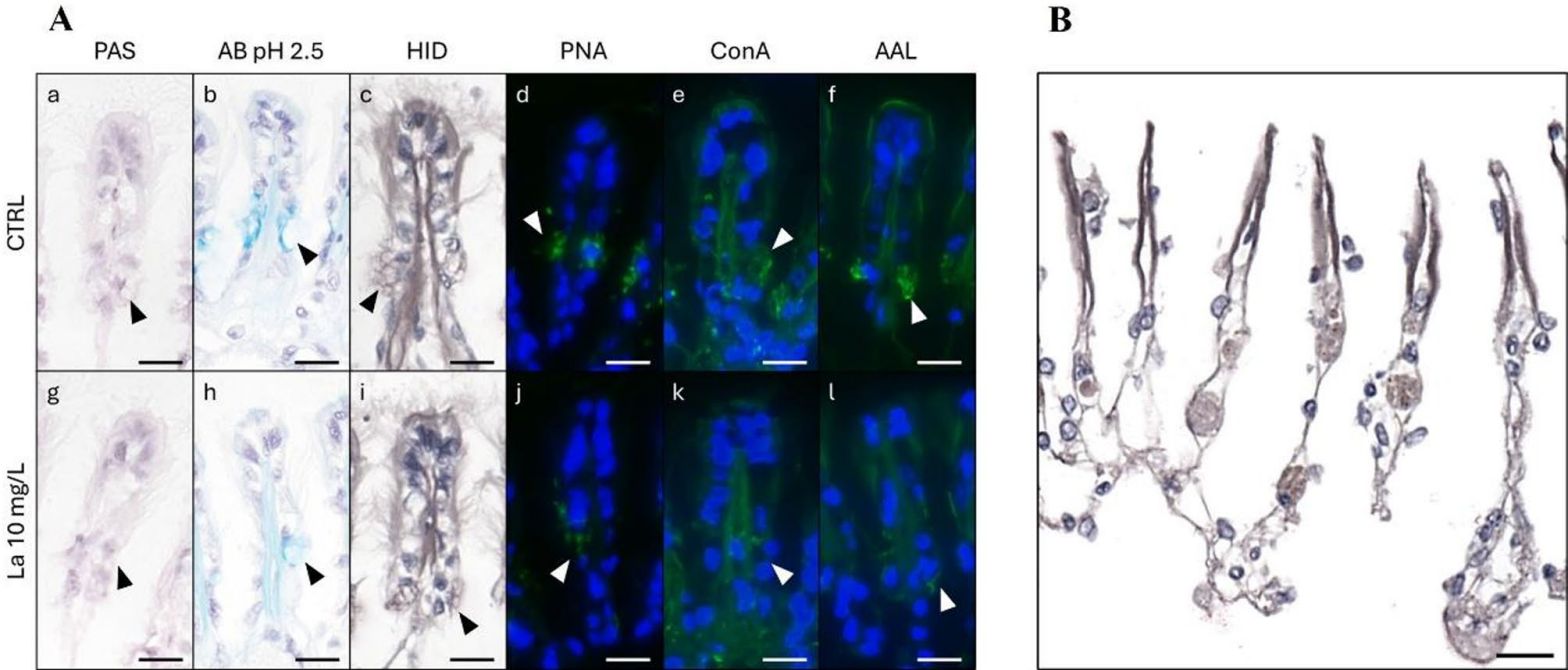
Histochemical Analysis. Part **A**: Histochemical and lectin-histochemical staining. The collar cells in the gill filament are indicated with arrowheads. Compared to the control group, a reduction in binding intensity is evident in the specimens treated with 10 mg/L La. PAS: Periodic Acid Schiff; AB pH 2.5: Alcian Blue pH 2.5. HID: High Iron Diamine; PNA: Lectin from *Arachis hypogaea* (peanut); ConA: Lectin from *Canavalia ensiformis* (Jack bean); AAL: Lectin from *Aleuria aurantia* (orange peel fungus). Part **B**: Specimen treated with 10 mg/L of La with necrotic areas and complete degeneration of the epithelial tissue. Nuclei are purple. Scale bars:10 μm.

A total of 20 specimens from the CTRL group and 18 specimens from the La10 group survived the three-day experimental period. The gills of both groups exhibited similar morphology; however, the CTRL group showed gills with a darker intensity of pink and a higher mucus content, whereas the La10 group had more transparent gills with less mucus. Histochemical analysis was performed on five CTRL samples and five La10 samples. In these samples, the gills were in good condition, characterized by long cilia and well-preserved collar cells within the gill filaments, as shown in Fig. 1-A, indicated with an arrowhead. Conventional histochemical analysis revealed a weaker PAS-positive reaction in the collar cells of both treatments. Alcian Blue-positive cells were detected in some specimens of both conditions (Fig. 1–A). Lectin binding was observed for PNA, ConA, and AAL. A reduction in binding intensity in the La10 group respect to the CTRL was observed.

HID staining was positive in only one specimen La10 revealing a notably foamy secretion in areas of necrosis (Fig. 1-B). Furthermore, in specimens treated with La10, a slight reduction in cilia height was observed, along with the presence of necrotic areas and, in some cases, complete degeneration of the epithelial tissue. (Fig. 1).

### Untargeted metabolomics analysis

The metabolic alterations in the gills of the treated groups were evaluated with an untargeted metabolomics analysis using ultra-high performance liquid chromatography coupled to high-resolution mass spectrometry (UHPLC-HRMS). Metabolites were extracted from the clam gills, using 15 gills from the CTRL group and 13 from the La10 group. For each specimen, shell width, total body weight, and gill weight were recorded (Table S2 in Supplementary Materials). We found no statistically significant differences between the CTRL and La10 groups in terms of length (*p* = 0.2216), total weight (*p* = 0.5456), or gill weight (*p* = 0.9727) (Table 1). The metabolite data for statistical analysis were normalized to gill weight.

**Table 1:** Morphometric measurements of clam samples prepared for metabolomic analysis, including mean (± SD) values for length (mm), total weight (g), and gill weight (g) in control (CTRL) and La10 groups *p*-values calculated with unpaired t test. * or Mann-Whitney^$^.

	CTRL	La10	*p*-value
Number of samples	15	13	
Length, mm [mean (SD)]	34.86 (± 2.75)	33.62 (± 2.50)	0.2216*
Total Weight, g [mean (SD)]	11.92 (± 3.26)	11.30 (± 1.80)	0.5456*
Gills Weight, g [mean (SD)]	0.21 (± 0.26)	0.14 (± 0.04)	0.9727^$^

**Fig. 2:**
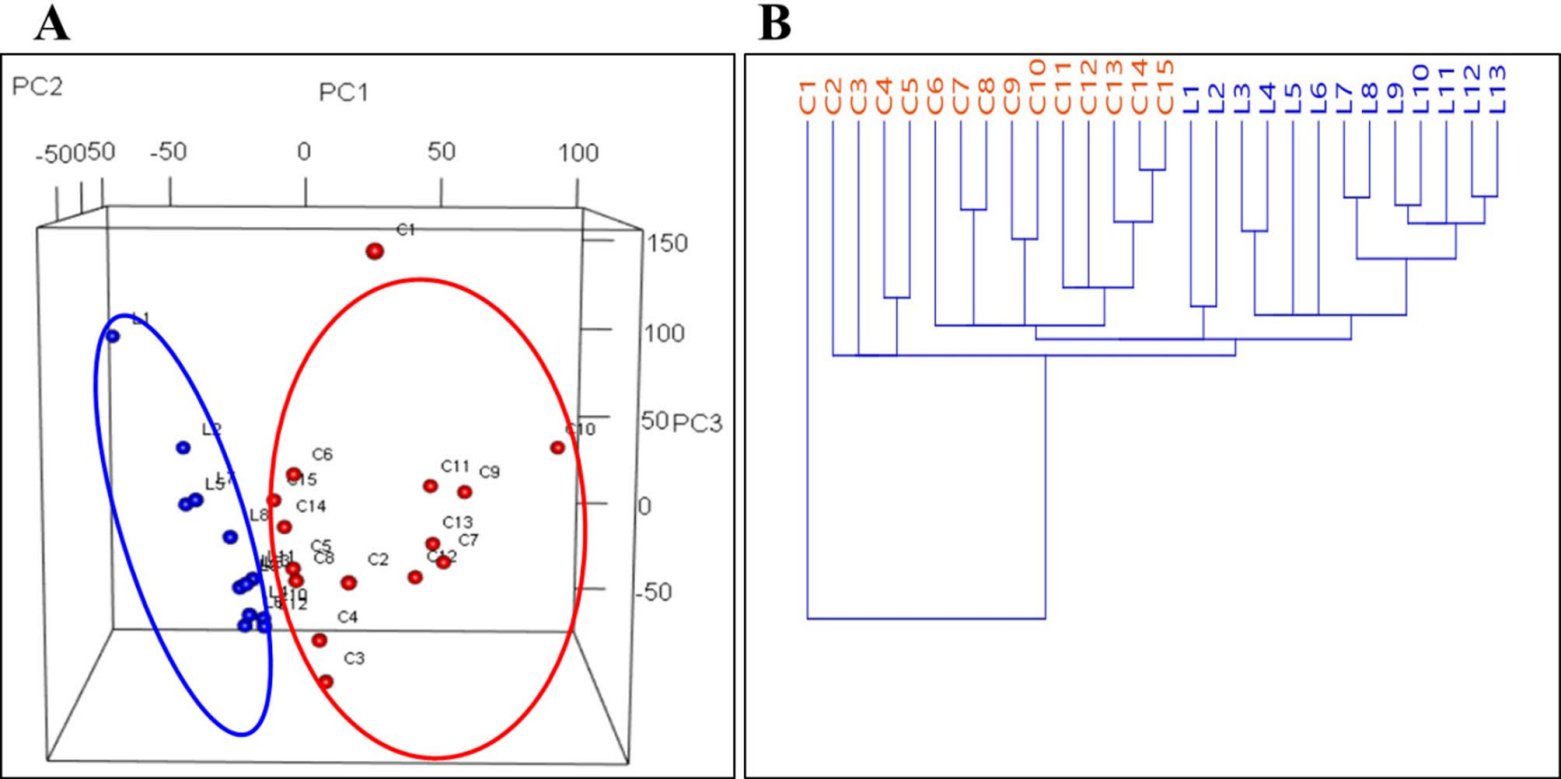
Principal Component Analysis. Part **A** displays the analysis of the first three principal components, where two distinct clusters are observed: controls in blue and treated samples in red. Part **B** illustrates the results of the Cluster Analysis: C1–C15 representing the CTRL samples, and L1–L13 corresponding to the La10 samples.

Raw data from the MS analysis were analyzed by CD software; approximately 6000 m/z features were identified after blank subtraction. Among these, 896 different m/z were successfully annotated. The number of annotated compounds was increased using MS2 and MS3 fragmentation data that CD is able to manage. Statistical processing revealed 188 metabolites that were differentially expressed between the La10 and CTRL samples. A total of 135 metabolites showed decreased concentrations in the treated samples, while 53 metabolites showed increased concentrations (Table S3 in Supplementary Materials). Principal Component Analysis (PCA), performed using Past 4.03, reveals that the first three principal components account for nearly 60% of the total variance in the dataset, effectively delineating two distinct clusters: CTRL (blue) and La10 (red), as shown in Fig. 2A. These results align with the cluster analysis based on Euclidean distance, which reveals that the La10 samples form a distinct cluster, separate from the CTRL group. Within the CTRL group, two subclusters are observed: one comprising samples C2 to C5, and the other including samples C6 to C15 (Fig. 2B). Notably, sample C1 displays a distinct clustering pattern compared to the other CTRL samples, reflecting natural biological variability.

**Fig. 3:**
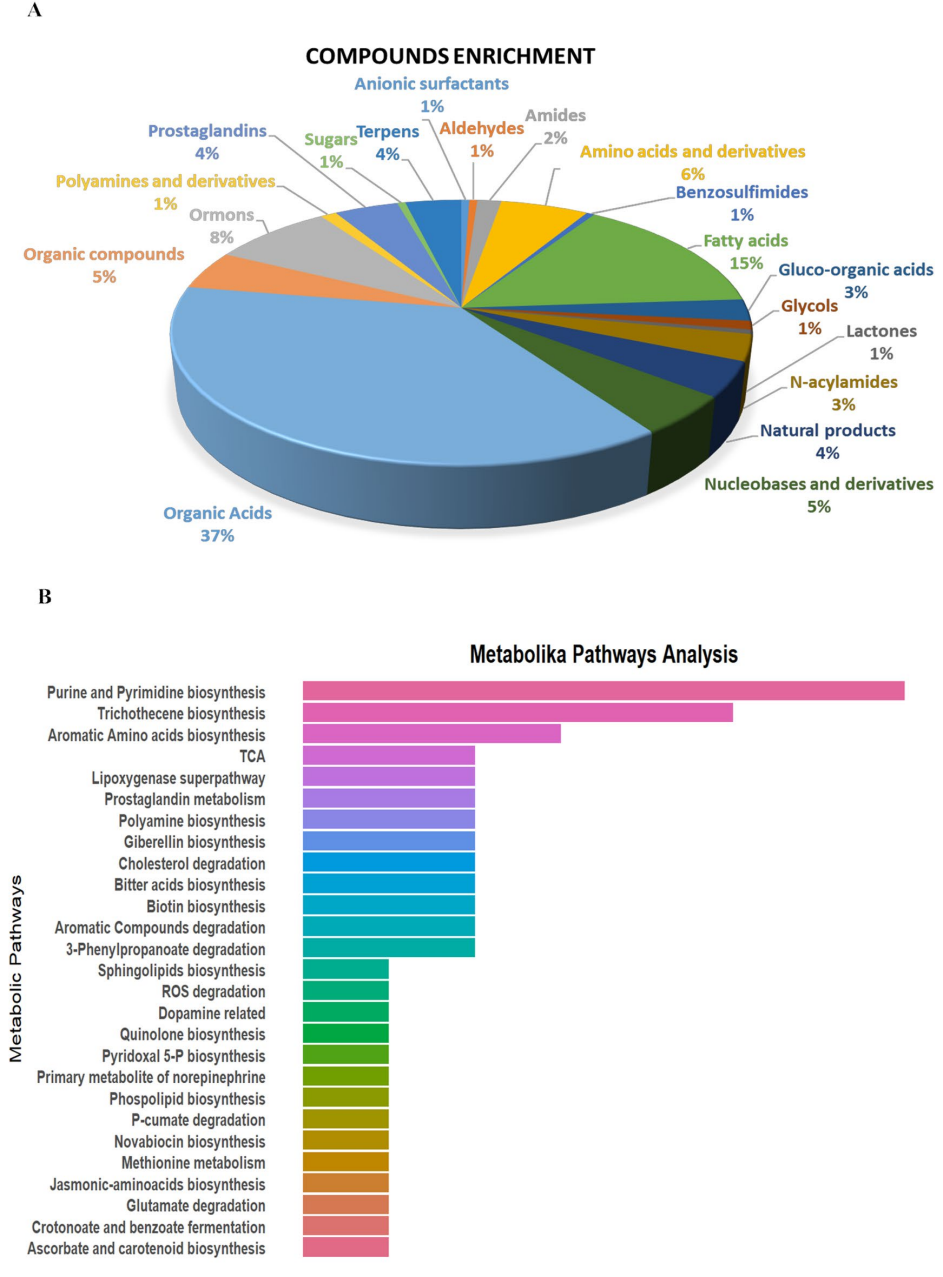
Metabolite enrichment analysis: Panel **A** displays a pie chart illustrating the distribution of metabolites across different chemical families, with their corresponding percentages. Panel **B** presents the pathways identified through metabolomic analysis.

The Compounds enrichment of DEMs, based on the classification in CD, indicated that over 50% were organic acids and fatty acids, followed by hormones, amino acids, nucleotides, and their derivatives (Fig. 3A). To map DEMs onto metabolic pathways, we used Metabolika, an internal tool of CD, (Fig. 3B). Pathways with the highest number of metabolites were related to the biosynthesis of purines and pyrimidines, aromatic amino acids and trichothecene.

**Fig. 4:**
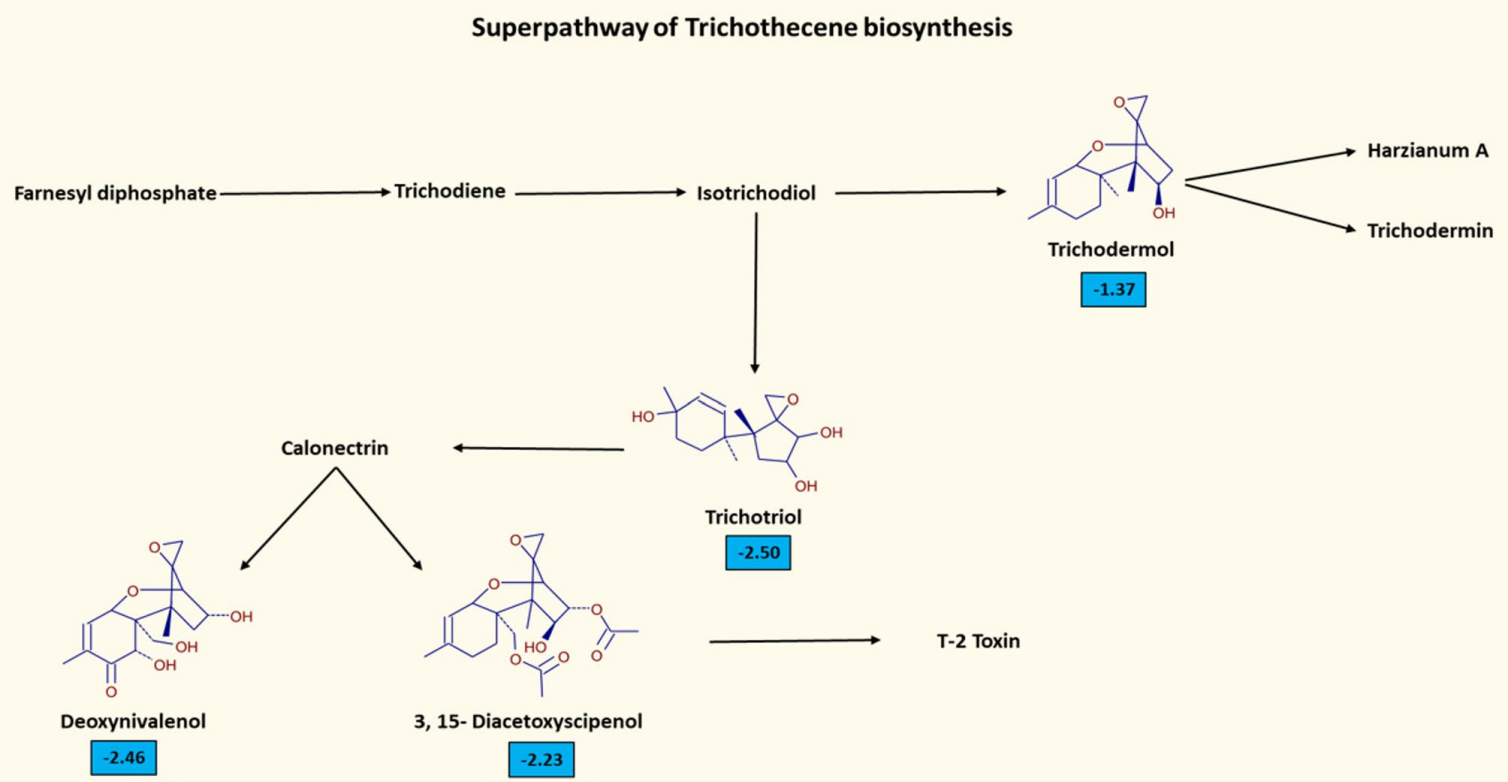
Trichothecene biosynthesis pathway. The figure shows the chemical structures of DEMs only in La treated samples. In blue are highlighted the Log2 Fold Change of the metabolites.

The decrease of metabolites associated with the Trichothecene biosynthesis pathway in the La treated gills (Fig. 4) suggests the decrease of mycotoxin synthesis, presumably due to the fungicidal activity of La^38^.

### IPA analysis

**Fig. 5:**
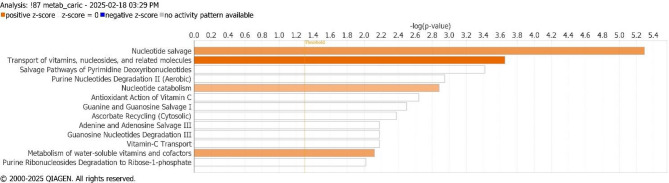
Enriched canonical pathways using differentially expressed metabolites in gills exposed to La. Enriched canonical pathways obtained by IPA. The bar chart displays pathway activation with a positive z- score highlighted in orange. White bars denote pathways with z-scores close to zero or those ineligible for analysis due to insufficient molecules in the dataset associated with the pathway. The standard IPA legend includes blue to represent negative z-scores; however, no inhibited pathways were predicted in this analysis, hence no blue bars are shown in the figure.

The IPA software enables the integration of molecular, disease, and biological process data from diverse experimental models into the Ingenuity Knowledge Base. This data is further validated through intensive literature curation to infer biological relationships and pathways. Although it may seem counterintuitive to combine relationships derived from model organisms, the approach is generally valid as many biological pathways are conserved across species, largely due to the preserved activity of many genes and proteins across various tissues and organisms^39^. IPA has been applied in various fields, including the study of environmental stress in marine organisms, where it helps identify metabolic pathways disrupted by pollutants^40^. In this study, IPA software was used to assess the biological significance of 188 metabolites that were differentially expressed between the La10 and CTRL samples. The analysis identified 64 key molecules (Table S4 in Supplementary Materials) involved 13 canonical pathways with a -log(p-value) > 2. Among these, 4 pathways exhibited a “positive z-score”, indicating the activation of pathways related to *Nucleotide Salvage*, *Transport of Vitamins and Nucleotides*, *Nucleotide Catabolism*, and the *Metabolism of Water-Soluble Vitamins and Cofactors* (Fig. 5). The network analyses shown in Fig. 5 was generated using Ingenuity Pathway Analysis (IPA) (QIAGEN Inc., https://digitalinsights.qiagen.com/products/ingenuity-pathway-analysis). These pathways also showed a notable overlap with the metabolite-related findings obtained from the Metabolika analysis, except for the Trichothecene biosynthesis pathway, which was missing from the list of IPA’s canonical pathways.

The analysis of “Categories and Diseases or Functions Annotation”, associated with DEMs, indicated that the activation of cell proliferation and replication across various cell types, as well as processes such as *Metabolism of hydrogen peroxide*, *Replication of RNA virus*, *Growth of bacteria* and *Concentration of ATP* (z score > 1) were the processes mainly involved in La treated samples. Conversely, the DEMs were also linked to inhibition of cell death in various tissue types and to a reduction in the *uptake of D-glucose* (z score < −1) (Table 2).

**Table 2:** Disease and functional categories of differentially expressed metabolites in gills exposed to la. Annotated disease and functional categories are listed along with their corresponding *p*-value, predicted activation state, activation z-score, and associated molecules.

Categories	Diseases or Functions Annotation	*p*-value	Predicted Activation State	Activation z-score	Molecules
Cell Death and Survival	Cell viability of tumor cell lines	0.00447	Increased	2.393	ascorbic acid, deoxyuridine, glutathione, oleic acid, testosterone, thymidine
Cellular Development, Cellular Growth and Proliferation	Cell proliferation of tumor cell lines	0.0155	Increased	1.679	ascorbic acid, glutathione, GMP, guanine, oleic acid, testosterone, thymidine
Free Radical Scavenging, Small Molecule Biochemistry	Metabolism of hydrogen peroxide	0.0094	Increased	1.274	ascorbic acid, glutathione, oleic acid, testosterone
Infectious Diseases, Organismal Injury and Abnormalities	Replication of RNA virus	0.00241	Increased	1.131	adenine, glutathione, guanine, heptanoic acid
Energy Production, Molecular Transport, Nucleic Acid Metabolism, Small Molecule Biochemistry	Concentration of ATP	0.0053	Increased	1.096	adenine, ascorbic acid, oleic acid, testosterone
Cellular Growth and Proliferation, Organismal Development	Growth of bacteria	0.0145	Increased	1.091	ascorbic acid, GMP, oleic acid, thymidine
Cellular Function and Maintenance	Metabolism of macromolecule	0.0288	Increased	1.082	ascorbic acid, glutathione, GMP, oleic acid, testosterone
Cell Cycle	Replication of cells	6.66E-06	Increased	1	adenine, guanine, testosterone, thymine
Cell Death and Survival, Organismal Injury and Abnormalities	Cell death of carcinoma cell lines	0.00435	Decreased	−2	ascorbic acid, glutathione, oleic acid, thymidine
Cancer, Cell Death and Survival, Organismal Injury and Abnormalities, Tumor Morphology	Necrosis of malignant tumor	0.00353	Decreased	−1.982	ascorbic acid, dodecanedioic acid, glutathione, oleic acid
Cell Death and Survival, Organismal Injury and Abnormalities	Cell death of lymphocytes	0.0053	Decreased	−1.677	ascorbic acid, glutathione, oleic acid, thymidine
Cell Death and Survival, Organismal Injury and Abnormalities	Cell death of connective tissue cells	0.0104	Decreased	−1	ascorbic acid, glutathione, oleic acid, thymidine
Carbohydrate Metabolism, Molecular Transport, Small Molecule Biochemistry	Uptake of D-glucose	0.0115	Decreased	−1	ascorbic acid, butyryl-L-carnitine, oleic acid, testosterone

**Fig. 6:**
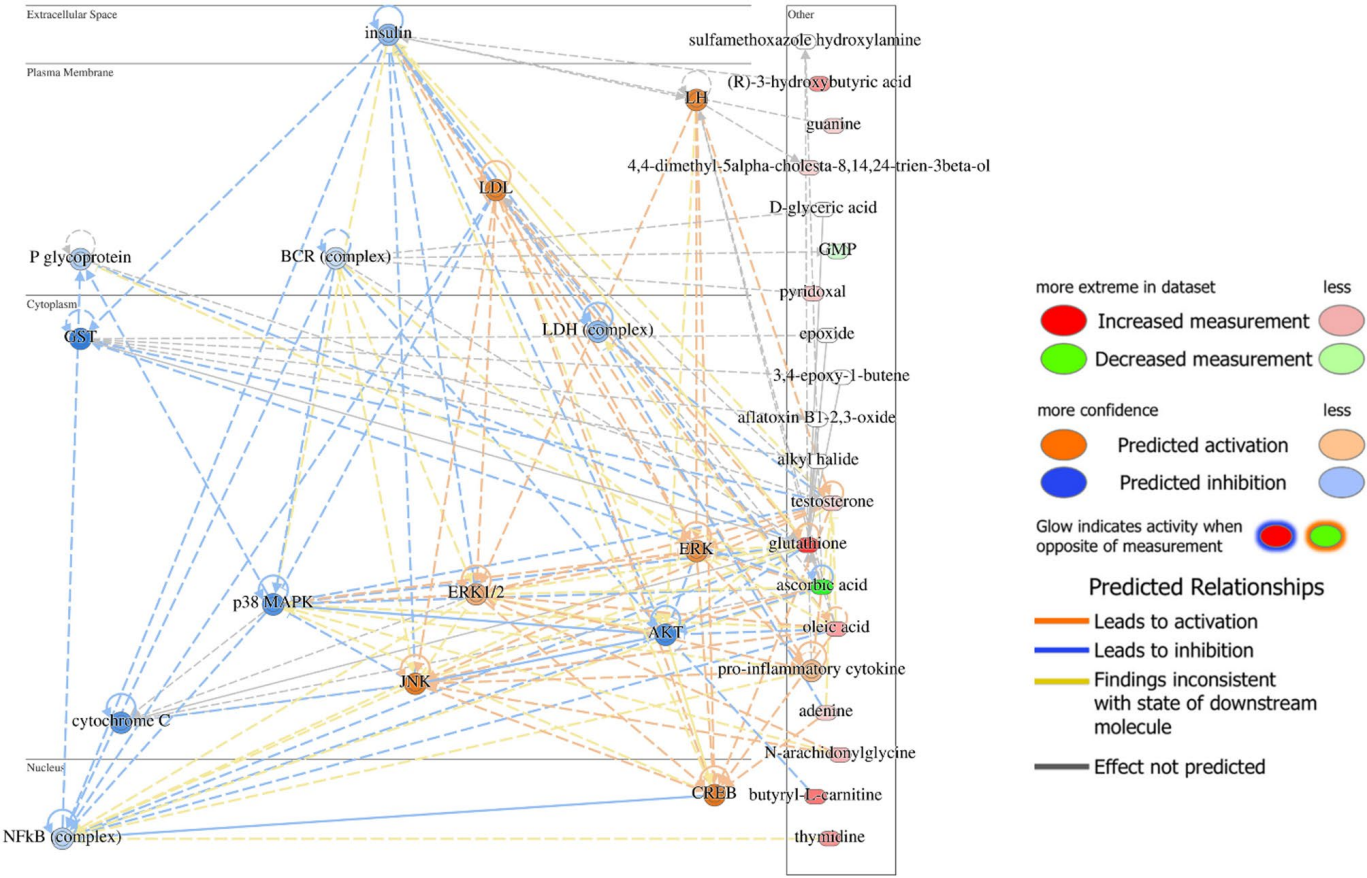
Network analysis containing DEMs in Gills exposed to La. The highest-ranking network highlights protein complexes predicted to be active (in orange) and those expected to be inhibited (in blue) across various cellular compartments.

The identified categories are mainly modulated by the overexpression of metabolites such as glutathione (GSH), oleic acid and thymidine, together with the downregulation of ascorbic acid. These metabolites among all the identified DEMs are probably more involved in the metabolic processes activated in the clam in presence of La. The networks analysis generated by IPA highlighted the highest-ranking network (score 34, 13 associated molecules) that included several compounds linked to cell growth and proliferation, infectious diseases, and tissue damage and abnormalities (Fig. 6).

The network analysis reveals a predicted activation of the ERK pathway in the cytoplasm, which is associated with the activation of the CREB signaling system in the nucleus. In contrast, the activities of Glutathione S-Transferase (GST) and cytochrome C in the cytoplasm, as well as P-glycoprotein (P-gp) at the plasma membrane, are downregulated. The network analyses shown in Fig. 6 was generated using Ingenuity Pathway Analysis (IPA) (QIAGEN Inc., https://digitalinsights.qiagen.com/products/ingenuity-pathway-analysis).

## Discussion

An integrated approach combining histopathological and metabolomic analyses reveals the effects of La exposure on Manila clams (*R. philippinarum*).

In specimens exposed to 10 mg/L of lanthanum (La10), a weak periodic acid–Schiff (PAS)-positive reaction was detected in the gill collar cells, indicating a low carbohydrate content. Alcian Blue (AB) staining yielded positive results in one control and two treated samples, suggesting potential alterations in acidic glycoconjugates. High Iron Diamine (HID) staining revealed the presence of foamy secretions in necrotic regions and complete epithelial tissue degeneration in treated specimens.

Furthermore, a marked reduction in the binding intensity of specific lectins—peanut agglutinin (PNA), concanavalin A (ConA), and *Aleuria aurantia* lectin (AAL)—was observed in La10-treated samples relative to controls. These findings indicate structural alterations in the glycosidic chains of mucin-secreting cells, specifically affecting galactosylated, mannosylated, and fucosylated residues. The reduced lectin binding suggests a compromised structural integrity and functionality of mucins, which play a critical role in gill protection and lubrication.

Using an untargeted metabolomic approach followed by statistical analysis, 188 metabolites were found to be differentially expressed between La10-treated and CTRL samples. An initial analysis of these DEMs using the Metabolika tool provided critical insights into the key metabolic pathways altered by La treatment, thereby enhancing the interpretative power of the metabolomic analysis. Metabolika tool has highlighted alterations in the Trichothecene biosynthesis superpathway. Trichothecenes constitute a large group of chemically related mycotoxins produced by fungi^41^ and share a central core structure consisting of a six-membered ring with a single oxygen atom, flanked by two carbon rings. This core also includes an epoxide group linking carbons 12 and 13, along with a double bond between carbons 9 and 10^42^. These two functional groups are mainly responsible for the ability of trichothecenes to inhibit protein synthesis and induce general cytotoxic effects^43^. The observed reduction of metabolites involved in this biosynthetic pathway suggests a potential fungicidal effect of La, as already reported in literature^38^, which should be further investigated through targeted studies evaluating its influence on fungal viability and activity.

Functional analysis pointed out four key metabolites among all DEMs: glutathione (GSH), oleic acid and thymidine, and ascorbic acid. These metabolites were mainly associated with disease and functional categories identified by IPA analysis. Notably, GSH and ascorbic acid are two critical antioxidants that protect cells from the harmful effects of oxidative stress^44–46^. Exposure to heavy metals like La has been shown to elevate GSH levels as a defensive mechanism against oxidative cellular damage^19^. The varying levels of GSH and ascorbic acid in clams exposed to La may serve as a defensive response, helping the organism mitigate cellular damage induced by oxidative stress. Oleic acid is a monounsaturated fatty acid found in various aquatic organisms, including mollusks^47^, and is the precursor of polyunsaturated fatty acids, which play a crucial role in the structure of cell membranes and energy production. In fact, alterations in membrane fatty acid composition are considered reliable biomarkers for assessing the nutritional quality of the food web^48,49^ and for monitoring toxicological effects on the aquatic biome^50,51^. Some studies indicate that the presence of organic pollutants in aquatic systems can alter fatty acid profiles by modifying lipid profiles^51,52^ and their profiles can be used as an indicator of stress for two marine bivalve species. Furthermore, other bivalve mollusks, such as *Mytilus edulis*, and *Mizuhopecten yessoensis*, when exposed to heavy metals like cadmium and copper, exhibit modifications in their lipid composition and fatty acid metabolism as a stress response^53,54^. The increased levels of oleic acid, observed in clams exposed to La, may result in an adaptive response to metal-induced stress, which triggers an upregulation of the production of reactive oxygen species (ROS), which has been shown to lead to lipid peroxidation^51^. Since oleic acid, a monounsaturated fatty acid, is less prone to peroxidation compared to polyunsaturated fatty acids, its relative increase may help maintain membrane integrity and fluidity under stress. Furthermore, exposure to heavy metals such as cadmium and 1-nonyl-4-phenol has been shown to induce metabolic changes in the microalga *Chlorella sorokiniana*, impacting both lipid and nucleotide metabolism. These changes lead to alterations in DNA repair mechanisms^55^. A similar effect could also be induced in clams exposed to La, where elevated levels of both thymidine and metabolites involved in the synthesis of pyrimidines and purines were detected (Table 2). The altered expression levels of GSH, ascorbic acid, oleic acid, and thymidine in clams exposed to La suggest a distinct metabolic stress response induced by the metal. These changes highlight their potential as biomarkers for La pollution.

The DEMs genera ted a network involving multiple compounds related to cell growth and proliferation, infectious diseases, and tissue damage or abnormalities. This network includes activation of the ERK pathway which plays a crucial role in many marine organisms in response to environmental stressors, including those induced by pollutants, by regulating key processes such as cell proliferation, differentiation, and survival^56^. Furthermore, this pathway is functionally expressed in clam gills^57^. The ERK system is noted to be related to the CREB system in the nucleus, which is also activated. The CREB (cAMP response element binding protein) system has been shown to be a cellular transcription factor in mammals^58^. It binds to certain DNA sequences called cAMP response elements (CREs), increasing or decreasing the transcription of downstream genes. In bivalve mollusk, the ERK-CREB signaling pathway has been found mainly activated in response to innate immune challenges^59,60^. The observed preference for ERK’s ability to activate CREB in the network may represent the clam’s response to trigger the transcription of genes associated with survival or the adaptation to stress induced by La. Among the systems predicted to be inhibited is the reduction in GST activity within the cytoplasm. This enzyme plays a crucial role in protecting cells from damage caused by oxidative stress, a condition that arises when there is an excessive accumulation of ROS^61^. Consequently, the reduction in GST activity in marine organisms can serve as a concerning indicator of physiological stress, cellular damage, and metabolic dysfunction, potentially signaling a compromise in detoxification and antioxidant defense mechanisms. In fact, studies have shown that exposure to hydrocarbons and other pollutants in bivalve mollusks leads to a decreased detoxification capacity and a reduction in GST activity, suggesting that these organisms become less capable of handling toxic compounds associated with marine pollution^62^. P-gp is also inhibited in plasma membrane. This is a membrane ATP-binding cassette transporter (ABC transporter) and plays a key role in the active transport of many molecules across cellular membranes. It is known to function in the efflux of toxic compounds, drugs, and metabolites from many cells, contributing to the protection of organisms from potentially harmful substances^63,64^. In the context of marine organisms and bivalves, there is some evidence that P-gp may play a role in defense against environmental contaminants, including heavy metals, pesticides, persistent organic pollutants, and other toxic compounds that accumulate in the marine environment^65^. Inhibition of P-gp in these organisms may, therefore, increase sensitivity to chemicals because when it is inhibited, the efflux of toxins decreases, leading to a higher concentration of contaminants within the organism. This may have ecological implications, as the organisms may be more susceptible to cellular damage and toxicity^66^. La appears to inhibit P-gp activity, which may pose a threat clam health by promoting the accumulation of contaminants within their tissues. La appears to inhibit Cytochrome C, a crucial protein in the mitochondrial electron transport chain, which plays a vital role in cellular respiration and apoptosis^67^. In marine organisms, particularly bivalves, cellular respiration is closely linked to the ability to adapt to changes in environmental conditions, such as variations in temperature, salinity or the presence of pollutants^68^. The inhibition of cytochrome c could therefore weaken the ability to respond to environmental stress, increasing vulnerability to environmental pollutants and reducing the ability of organisms to adapt.

Inhibition of P-gp, GST and the associated accumulation of GSH are the main components of the multi-xenobiotic resistance (MXR) mechanism. In this context, GST catalyzes the conjugation of reduced GSH to xenobiotics, resulting in less toxic, more water soluble compounds^69^. This conjugate is then transported out of the organism by specific proteins These conjugates are subsequently transported out of the cell by ATP-binding cassette (ABC) transporter such as P-gp^70^. Therefore, we may postulate that La acts as a GST inhibitor, preventing the formation of GSH conjugates, leading to a buildup of GSH inside the cell (Fig. 6). Furthermore, inhibition of P-gp may limit the flux of GSH conjugates, contribution to their intracellular retention. Together, the inhibition of GST and P-gp may significantly compromise the MXR system and detoxification capacity in bivalves. The combined inhibitory effects of La on key proteins such as GST, P-gp, and Cytochrome suggest a broader disruption of detoxification with potential ecotoxicological implications for the health and survival of bivalves but also the broader marine populations.

## Conclusions

La exposure induces significant changes in the carbohydrate composition and glycosidic chains of mucins in the gills of Manila clams. These alterations are linked to necrosis, epithelial tissue degeneration, and reduced mucus secretion, potentially impairing the gills’ protective function. Such effects underscore the cellular and tissue damage caused by La, with serious implications for clam health and their ability to withstand environmental stress. Additionally, metabolic analysis revealed altered expressions of four key metabolites: GSH, aspartic acid, oleic acid, and thymidine, which may serve as indicators of oxidative stress and DNA damage induced by La at the gill level. Overall, La exposure does not only threaten clam health through metabolic disruption but may also have broader ecological consequences for marine ecosystems.

## Supplementary Information

Below is the link to the electronic supplementary material.

Supplementary Material 1

## Data Availability

The raw data generated and analyzed during the current study are available in the MetaboLights repository with the number identifier MTBLS12649 [https://www.ebi.ac.uk/metabolights/MTBLS12649].
