# Supplementary material for: Lanthanum exposure and its metabolomic effects on Ruditapes philippinarum
Source: Sci Rep. 2025 Sep 26;15:33166. doi: 10.1038/s41598-025-15576-7 (PMC12475223; doi:10.1038/s41598-025-15576-7)
Supplement: Supplementary file 1 — Supplementary Material 1 [file 41598_2025_15576_MOESM1_ESM.pdf]

Supplementary materials

Table S1: Lectins used in histochemical investigations. The Buffer used in dilutions is Hepes.

	**Binding sites**	**Dilution mg/ml**	**Inhibitory sugar concentration**
PNA: *Arachis hypogaea*	Galβ1,3GalNAc	10	0.2 M Gal
SBA: *Glycine max*	GalNAc	20	0.2 M GalNAc
WGA: *Triticum vulgaris*	(GlcNAcβ1,4)n	20	0.5 M GlcNAc
LTA: *Tetragonolobus purpureus*	L-Fucα1,6GlcNAc; L-Fucα1,2Galβ1,4[LFuc1,3]GlcNAcβ1,6R	20	0.2 M L-Fuc
UEA I: *Ulex europaeus*	Fucα1,2	10	0.2 M L-Fuc
AAL: *Aleuria aurantia*	Fucα1,6GlcNAc-βNAsn; Fucα1,3,Fucα1,4	10	0.2 M L-Fuc
SNA: *Sambucus nigra*	Neu5Acα2,6Gal/GalNAc	20	0.2 M Neu5Ac
MAA II: *Maackia amurensis*	Neu5Acα2,3Galβ1,4GlcNAc	20	0.2 M Neu5Ac
ConA: *Canavalia ensiformis*	D-Man, D-Glc	20	0.1 M MαM

Table S2: Samples prepared for metabolomic analysis include measurements of length, total weight, and gill weight for each clam sample

Sample	Case	Length	Total Weight	Gills Weight
C1	CTRL	31	9.04	0.07
C2	CTRL	39	16.45	0.16
C3	CTRL	37	12.67	0.3
C4	CTRL	36	16.02	0.21
C5	CTRL	37	14.21	0.15
C6	CTRL	37	12.45	0.11
C7	CTRL	34	10.23	0.15
C8	CTRL	40	19.49	0.14
C9	CTRL	32	9.94	0.11
C10	CTRL	33	9.61	0.11
C11	CTRL	35	10.32	0.12
C12	CTRL	32	9.34	0.16
C13	CTRL	33	9.87	1.14
C14	CTRL	32	8.28	0.14
C15	CTRL	35	10.88	0.13
L1	La10	31	9.45	0.08
L2	La10	30	9.25	0.11
L3	La10	38	13.86	0.15
L4	La10	37	13.65	0.18
L5	La10	34	11.08	0.13
L6	La10	36	12.03	0.23
L7	La10	35	13.82	0.12
L8	La10	33	12.76	0.13
L9	La10	34	10.56	0.13
L10	La10	33	10.13	0.17
L11	La10	34	11.68	0.14
L12	La10	32	9.02	0.17
L13	La10	30	9.56	0.14

.

Table S3: The table displays the differentially expressed metabolites between control and treated samples. Metabolites with decreased concentration in La10 samples are highlighted in blue, while those with increased concentration are shown in red. For each metabolite, the name, formula, PubChem Compound ID (CID) number, molecular weight, Log2 Fold Change (FC), and p-value are provided.

Name	Formula	CID number	Calc. MW	Log2FC	p-value
Ethanolamine	C2 H7 N O	700	61,0527	1,40	4,05E-03
3-Buten-2-one	C4 H6 O	6570	70,0418	-1,73	1,77E-03
Sulfite	H2 O3 S	1099	81,9723	1,23	4,87E-03
Pyruvate	C3 H4 O3	107735	88,0161	-1,48	3,88E-04
Butanoate	C4 H8 O2	104775	88,0524	-1,78	5,06E-03
Sorbaldehyde	C6 H8 O	637564	96,0575	-1,43	2,96E-03
5-Valerolactone	C5 H8 O2	10953	100,0525	-1,16	3,67E-03
4-Aminobutanoate	C4 H9 N O2	5460232	103,0634	1,05	3,61E-03
(R)-3-Hydroxybutanoate	C4 H8 O3	92135	104,0474	2,58	1,38E-02
Pyridyl-3-methanol	C6 H7 N O	7510	109,0527	1,38	4,97E-03
Sorbic acid	C6 H8 O2	643460	112,0525	1,10	3,91E-02
Hydrosorbic acid	C6 H10 O2	5282708	114,0681	-1,99	1,91E-03
L-2,4-Diaminobutanoate	C4 H10 N2 O2	134490	118,0743	-1,28	1,30E-02
6-Amino-m-cresol	C7 H9 N O	76082	123,0684	1,14	2,29E-02
Thymine	C5 H6 N2 O2	1135	126,0431	1,49	2,95E-03
(1S,2R)-3-Methylcyclohexa-3,5-diene-1,2-diol	C7 H10 O2	440399	126,0681	-1,82	2,95E-03
Cyclohexane-1-carboxylate	C7 H12 O2	7413	128,0839	-1,44	5,92E-03
Heptylic acid	C7 H14 O2	8094	130,0995	-1,88	3,79E-03
2-hydroxycaproicacid	C6 H12 O3	99824	132,0786	-1,33	1,34E-02
Adenine	C5 H5 N5	190	135,0545	1,13	1,19E-04
(2E,4E,6Z)-2,4,6-Octatrienoic acid	C8 H10 O2	11491993	138,0680	-1,28	2,09E-02
7-oxoheptanoate	C7 H12 O3	9548029	144,0787	-1,57	7,38E-03
erythro-3-Hydroxy-L-aspartate	C4 H7 N O5	14463	149,0325	-1,73	4,23E-02
Guanine	C5 H5 N5 O	135398634	151,0494	1,02	9,54E-03
3-Sulfinopyruvate	C3 H4 O5 S	110	151,9779	-1,66	2,73E-03
3-Oxoadipate	C6 H8 O5	5459800	160,0372	-2,99	2,73E-04
Pimelate	C7 H12 O4	385	160,0730	-2,11	2,24E-03
D-Alanyl-D-alanine	C6 H12 N2 O3	5460362	160,0848	-1,11	3,88E-02
3-(3-Hydroxyphenyl)propanoate	C9 H10 O3	6931501	166,0629	-1,08	2,16E-03
Pyridoxal	C8 H9 N O3	1050	167,0582	1,29	4,00E-02
Germicidin H	C9 H12 O3	132543964	168,0786	-3,09	1,17E-03
3,4-Dihydroxyphenylglycol	C8 H10 O4	91528	170,0580	-1,09	6,68E-03
Dendryphiellic acid B	C9 H14 O3	96009607	170,0943	-1,84	2,29E-03
p-Toluenesulfonic acid	C7 H8 O3 S	6101	172,0195	-2,05	1,12E-02
cis-4-octenedioic acid	C8 H12 O4	11805205	172,0736	-1,26	4,73E-04
4,7-dioxo-octanoic acid	C8 H12 O4	244084	172,0736	-1,58	7,18E-03
9-Oxo-nonanoate	C9 H16 O3	129745248	172,1099	-1,67	1,46E-03
N-Acetyl-L-glutamate 5-	C7 H11 N O4	192878	173,0688	-1,03	3,84E-02

semialdehyde					
4-phenolsulfonic acid	C6 H6 O4 S	4765	173,9987	1,19	2,66E-02
cis-Aconitate	C6 H6 O6	643757	174,0164	-2,03	7,33E-04
Shikimate	C7 H10 O5	8742	174,0528	-1,97	1,98E-03
Subericacid	C8 H14 O4	84204	174,0892	-1,72	1,09E-03
L-Ascorbate	C6 H8 O6	54670067	176,0320	-4,73	1,02E-03
6,8-dihydroxy-octanoic acid	C8 H16 O4	5312790	176,1050	-2,36	5,50E-03
Salsolinol	C10 H13 N O2	91588	179,0946	-2,11	4,83E-03
4-Hydroxyphenylpyruvate	C9 H8 O4	979	180,0424	-1,41	1,40E-03
Stipitatate	C8 H6 O5	20501	182,0216	-1,81	2,07E-03
saccharin	C7 H5 N O3 S	5143	182,9991	-2,78	1,46E-03
3-(5,6-Dihydroxycyclohexa-1,3-dien-1-yl)propanoate	C9 H12 O4	9543127	184,0735	-1,49	1,87E-03
5-oxo-7E-decenoic acid	C10 H16 O3	5312883	184,1100	-1,28	1,09E-03
8-Amino-7-oxononanoate	C9 H17 N O3	5460197	187,1209	-1,96	3,94E-04
p-Cresylsulfate	C7 H8 O4 S	4615423	188,0143	1,10	4,70E-02
3-Butene-1,2,3-tricarboxylic acid	C7 H8 O6	592363	188,0323	-1,42	4,27E-04
Azelaicacid	C9 H16 O4	119967	188,1049	-1,61	1,00E-03
3-Dehydroquinate	C7 H10 O6	5460271	190,0478	-1,66	2,39E-02
(2E,6E,10E)-2,6,10-Dodecatrienoic acid	C12 H18 O2	11966271	194,1307	-1,14	3,33E-03
1-Thio-beta-D-glucopyranose	C6 H12 O5 S	444809	196,0402	-1,49	6,34E-03
11-oxo-undeca-5,8-dienoic acid	C11 H16 O3	134812119	196,1099	-1,13	4,66E-03
cis-2,3-Dihydroxy-2,3-dihydro-p-cumate	C10 H14 O4	9543088	198,0892	-2,47	1,85E-03
Cladosporacid E	C10 H16 O4	156581614	200,1049	-3,10	1,41E-03
Capryloylglycine	C10 H19 N O3	84290	201,1365	-2,52	8,57E-04
Sebacicacid	C10 H18 O4	5192	202,1205	-2,25	1,94E-03
3,10-dihydroxydecanoic acid	C10 H20 O4	9859090	204,1361	-2,56	4,98E-03
3-Dimethylallyl-4-hydroxybenzoate	C12 H14 O3	54746231	206,0943	-1,35	6,01E-03
Garcinia acid	C6 H8 O8	185620	208,0219	-1,99	2,15E-02
a Jasmonic acid	C12 H18 O3	25245888	210,1256	-1,66	2,40E-03
3-[(3aS,4S,5R,7aS)-5-Hydroxy-7a-methyl-1-oxo-octahydro-1H-indene-4-carboxylate	C11 H16 O4	90658696	212,1049	-1,94	1,79E-03
Traumatin	C12 H20 O3	5312889	212,1412	-1,94	1,95E-03
Pantheric Acid C	C11 H18 O4	145721313	214,1205	-1,71	2,25E-03
Cephalosporolide J	C10 H16 O5	21630881	216,0994	-1,50	6,16E-03
Undecanedioic acid	C11 H20 O4	15816	216,1361	-1,88	2,64E-03
12-hydroxyjasmonic acid	C12 H18 O4	5497122	226,1205	-1,75	1,42E-03
2'-Deoxyuridine	C9 H12 N2 O5	13712	228,0742	1,43	4,76E-02
Dodecanedioicacid	C12 H22 O4	12736	230,1518	-3,01	2,49E-03
Butyryl-L-carnitine	C11 H21 N O4	213144	231,1471	2,98	4,21E-02
3-[(3aS,4S,7aS)-7a-Methyl-1,5-dioxo-octahydro-1H-inden-4-yl]propanoate	C13 H18 O4	15944652	238,1205	-1,18	6,10E-03
3-carboxy-4-methyl-5-propyl-2-furanpropanoic acid	C12 H16 O5	123979	240,0999	-1,51	3,68E-03

Penicillone	C11 H14 O4 S	23651016	242,0613	-3,42	7,99E-04
Thymidine	C10 H14 N2 O5	5789	242,0900	2,44	2,24E-02
Glycerophosphoglycerol	C6 H15 O8 P	439964	246,0505	-3,34	1,93E-03
3-hydroxy-dodecanedioic acid	C12 H22 O5	16663321	246,1467	-2,83	2,51E-03
ascr#9	C11 H20 O6	71514783	248,1259	-2,91	1,03E-04
Trichodermol	C15 H22 O3	12315016	250,1568	-1,43	2,41E-03
Glutaurine	C7 H14 N2 O6 S	68759	254,0572	-1,13	2,82E-02
Northienamycin	C10 H14 N2 O4 S	132448	258,0674	1,93	3,41E-03
Tensyuic acid B	C12 H18 O6	23651873	258,1103	-1,42	5,33E-03
(Z)-2-(7-hydroxyoctyl)pent-2-enedioic acid	C13 H22 O5	101893003	258,1468	-1,95	5,13E-03
2-Heptyl-3-hydroxy-4(1H)-quinolone	C16 H21 N O2	2763159	259,1572	-1,59	6,38E-03
ascr#12	C12 H22 O6	86289663	262,1416	-2,79	3,85E-04
7-mercaptoheptanoylthreonine	C11 H21 N O4 S	24892802	263,1192	3,44	1,90E-02
4-Prenylphlorisobutyrophenone	C15 H20 O4	25203134	264,1362	-2,27	2,33E-03
Separacene A	C15 H22 O4	132607460	266,1519	-2,29	2,56E-03
Dodecyl sulfate	C12 H26 O4 S	8778	266,1551	-1,09	3,20E-03
Norspermidine-2,3-dihydroxybenzoate	C13 H21 N3 O3	90657820	267,1584	-1,04	3,33E-03
Peniciisocoumarin B	C14 H20 O5	145720895	268,1311	-3,10	1,91E-03
Trichotriol	C15 H24 O4	184134	268,1675	-2,50	3,17E-03
Pentadecanedioic acid	C15 H28 O4	160576	272,1987	-2,35	2,17E-03
3-hydroxy-tetradecanedioic acid	C14 H26 O5	20848956	274,1780	-4,75	2,03E-03
Daumone	C13 H24 O6	11471380	276,1573	-3,74	2,33E-03
Pantetheine	C11 H22 N2 O4 S	439322	278,1300	2,33	9,34E-03
4-Prenylphlorisovalerophenone	C16 H22 O4	13411740	278,1519	-1,26	4,52E-03
(1R,2R,5R,9R,10S,13R)-13-Hydroxy-5,9,13-trimethyl-3,14,15-trioxatetracyclo[8.5.0.0~1,6~.0~2,12~]pentadecan-4-one	C15 H22 O5	5144683	282,1468	-3,62	2,06E-03
Oleate	C18 H34 O2	445639	282,2559	2,09	3,79E-02
Tensyuic acid E	C14 H22 O6	23652018	286,1418	-4,00	6,30E-04
N1_N12-Diacetylspermine	C14 H30 N4 O2	132680	286,2369	3,52	1,00E-02
Prosopinine	C16 H33 N O3	42608371	287,2461	-1,50	5,06E-03
Testosterone	C19 H28 O2	6013	288,2090	1,12	1,05E-02
10_16-Dihydroxyhexadecanoicacid	C16 H32 O4	441449	288,2301	-2,43	1,92E-03
ascr#14	C14 H26 O6	86289672	290,1730	-3,87	1,83E-03
N6-Dimethyladenosine	C12 H17 N5 O4	440004	295,1281	2,48	2,49E-02
Deoxynivalenol	C15 H20 O6	40024	296,1261	-2,46	1,93E-03
Tetranor-PGE1	C16 H26 O5	6449794	298,1781	-3,70	2,59E-03
Norethindrone	C20 H26 O2	6230	298,1933	1,07	1,14E-02
Nonadecanoicacid	C19 H38 O2	12591	298,2873	-2,15	3,17E-05
N-(3-hydroxy-dodecanoyl)-homoserine lactone	C16 H29 N O4	10891902	299,2097	1,77	4,53E-03
3-Dehydrosphinganine	C18 H37 N O2	439853	299,2825	2,14	4,74E-02
Tetranor-PGF1alpha	C16 H28 O5	52921878	300,1936	-2,08	3,48E-03
2-methyl-hexadecanedioic acid	C17 H32 O4	9543663	300,2300	1,18	3,34E-02

9-hydroxy-hexadecan-1,16-dioic acid	C16 H30 O5	16061042	302,2093	-4,07	1,94E-03
ascr#10	C15 H28 O6	86289662	304,1886	-2,95	2,86E-03
3alpha_11beta-Dihydroxy-5alpha-androstane-17-one	C19 H30 O3	10286365	306,2195	1,38	1,09E-02
Glutathione	C10 H17 N3 O6 S	124886	307,0838	4,51	2,99E-02
Ethyl oleate	C20 H38 O2	5363269	310,2872	1,83	1,27E-03
2S-hydroxy-3-(10Z-tetradecenoyloxy)-propanoic acid	C17 H30 O5	73242181	314,2093	-2,68	3,27E-03
Gibberellin A9	C19 H24 O4	5281984	316,1674	-2,35	7,28E-03
Kauralexin B3	C20 H28 O3	90658844	316,2039	1,23	4,93E-03
bhas#10	C15 H28 O7	86289814	320,1838	-4,40	1,53E-03
Butyl 3-O-beta-D-glucopyranosyl-butanoate	C14 H26 O8	56936277	322,1629	-3,81	3,68E-03
4,7,10,13-Docosatetraynoic acid	C22 H28 O2	9543595	324,2090	1,20	1,04E-04
Heneicosylic acid	C21 H42 O2	16898	326,3184	-1,26	4,82E-03
N-palmitoyl alanine	C19 H37 N O3	14961184	327,2774	1,44	4,03E-02
(9S,10E,12S,13S,15Z)-9,12,13-Trihydroxy-10,15-octadecadienoic acid	C18 H32 O5	10936354	328,2249	-1,99	1,80E-03
Tetranor-PGFM	C16 H26 O7	3246853	330,1679	-2,19	4,15E-03
Gibberellin A20	C19 H24 O5	5280481	332,1625	-1,90	1,48E-02
Isopentenyladenosine	C15 H21 N5 O4	24405	335,1595	2,72	1,21E-02
LMST02020108	C19 H30 O5	146134	338,2093	-1,33	6,76E-03
Docosanamide	C22 H45 N O	76468	339,3501	-2,36	1,04E-02
11-dehydro-2,3-dinor-TXB2	C18 H28 O6	35024530	340,1887	-3,51	2,09E-03
(10R,13S,17R)-10,13-Dimethyl-5,6,7,8,9,10,11,12,13,14,15,16-dodecahydro-3'H-spiro[cyclopenta[a]phenanthrene-17,2'-furan]-3,5'(4H,4'H)-dione	C22 H30 O3	23215534	342,2195	1,15	4,60E-04
8-Epiiridotrial glucoside	C16 H24 O8	155942	344,1469	-1,18	5,79E-04
delta10-13-PhytoF	C18 H32 O6	126457311	344,2198	-1,32	1,90E-03
(+/-)-14-HDoHE	C22 H32 O3	11566378	344,2350	1,12	1,21E-02
Anandamide (20:5, n-3)	C22 H35 N O2	5283450	345,2667	2,02	1,50E-03
IMP	C10 H13 N4 O8 P	135398640	348,0470	-1,36	2,34E-02
LMST02030199	C21 H32 O4	44263347	348,2300	1,06	4,63E-03
Resocortol	C22 H32 O4	20055396	360,2300	1,12	7,22E-04
1-O-(2R-hydroxy-octadecyl)-sn-glycerol	C21 H44 O4	137323865	360,3240	-2,36	5,99E-05
N-arachidonoyl glycine	C22 H35 N O3	5283389	361,2618	1,74	7,08E-03
24:3(15Z,18Z,21Z)	C24 H42 O2	52921854	362,3181	2,16	9,74E-03
GMP	C10 H14 N5 O8 P	135398631	363,0580	-1,07	1,19E-02
dhas#18	C17 H32 O8	137628431	364,2100	-3,84	1,06E-03
3,15-Diacetoxyscirpenol	C19 H26 O7	102515232	366,1678	-2,23	2,72E-03
(3R,5R)-3-O-β-D-mannosyl-3,5-dihydrodecanoic acid	C16 H30 O9	CHEBI:33563 /atlas 8022	366,1889	-3,41	2,57E-03
TXB3	C20 H32 O6	5283140	368,2199	-1,18	3,03E-03
6-keto-PGF1alpha	C20 H34 O6	5280888	370,2356	-1,94	1,15E-03

N-arachidonoyl alanine	C23 H37 N O3	40846579	375,2774	1,51	2,16E-03
PA(14:0/0:0)	C17 H35 O7 P	9547180	382,2120	1,32	1,25E-05
N-palmitoleyl glutamine	C21 H38 N2 O4	100937253	382,2833	3,26	2,16E-04
12-hydroxyjasmonic acid 12-O-beta-D-glucoside	C19 H30 O8	11966210	386,1942	-3,38	1,03E-03
methyl 9-hydroperoxy-10,12,13,15-bisepidioxy-16E-octadecenoate	C19 H32 O8	5282886	388,2099	-5,05	6,92E-04
17-phenyl-trinor-PGF2alpha	C23 H32 O5	5283081	388,2247	-2,01	5,78E-03
1-O-(2R-hydroxy-eicosanyl)-sn-glycerol	C23 H48 O4	137323867	388,3552	-1,19	5,70E-03
(-)-11-hydroxy-9,10-dihydrojasmonic acid 11-beta-D-glucoside	C18 H30 O9	11966292	390,1891	-2,66	6,97E-04
Ergosta-5,7,22,24(28)-tetraen-3-β-ol	C28 H42 O	129682671	394,3235	1,03	5,38E-03
4Z,7Z,10Z,13Z,16Z,19Z,22Z,25Z-octacosaoctaenoic acid	C28 H40 O2	52921798	408,3028	1,19	4,51E-02
4,4-Dimethyl-cholesta-8,14,24-trienol	C29 H46 O	443212	410,3549	1,01	5,87E-03
methyl 5-hydroperoxy-6,8,9,11-bisepidioxy-12,14-eicosadienoate	C21 H34 O8	5282894	414,2254	-3,00	1,25E-03
Ascorbyl palmitate	C22 H38 O7	54680660	414,2623	-2,59	2,05E-03
1,4-bis[(2-ethylhexyl)oxy]-1,4-dioxobutane-2-sulfonic acid	C20 H38 O7 S	11339	422,2339	-2,28	1,42E-02
Sarcoehrendin E	C23 H36 O7	5280888	424,2461	-1,78	2,85E-03
LMST04010114	C24 H40 O6	5283893	424,2826	-1,84	6,47E-03
Coprisidin B	C22 H19 N O8	132526506	425,1105	-3,04	7,86E-03
S-glutathionyl-L-cysteine	C13 H22 N4 O8 S2	10455148	426,0880	-1,13	1,28E-02
2-glyceryl-PGD2	C23 H38 O7	24778486	426,2618	-2,18	1,30E-03
Dihomo-gamma-linolenoyl dopamine	C28 H43 N O3	16759349	441,3243	1,53	3,66E-03
2-(Dodecanoylamino)octyl 2-hydroxyethyl hydrogen phosphate	C22 H46 N O6 P	11744548	451,3061	1,14	1,76E-03
18-acetoxy-PGF2alpha-11-acetate	C24 H38 O8	5283090	454,2566	-2,59	2,74E-03
(+)-24-Dammarene-3alpha,12beta,20S-triol	C30 H52 O3	42608288	460,3916	1,44	1,93E-04
Certonardosterol J	C29 H50 O4	21629553	462,3708	1,01	2,91E-03
PE(P-19:1(12Z)/0:0)	C24 H48 N O6 P	42607471	477,3217	1,33	4,41E-04
11-beta-hydroxyandrosterone-3-glucuronide	C25 H38 O9	53480452	482,2514	-1,89	3,54E-03
Cer(m18:0/16:0)	C34 H69 N O2	134812119	523,5327	-1,07	2,02E-02
LMST05040006	C29 H51 N O7 S	42608424	557,3398	-1,21	3,83E-03
1-(2-methoxy-eicosanyl)-sn-glycero-3-phosphoserine	C27 H56 N O9 P	137323955	569,3694	-1,03	3,86E-02
PE(18:4(6Z,9Z,12Z,15Z)/22:6(4Z,7Z,10Z,13Z,16Z,19Z))	C45 H70 N O8 P	96009607	783,4828	-1,76	2,70E-03
N-Acetyl-L-leucyl-L-alanyl-L-leucyl-L-lysyl-L-tyrosyl-L-leucyl-L-alanyl-L-threonyl-L-prolyl-L-valinamide	C55 H92 N12 O13	145957770	1128,6915	1,31	9,23E-03

Table S4: The table displays the 64 molecules identified by the IPA bioinformatics software. Highlighted in orange the molecules involved in multiple metabolic processes. For each metabolite, the table includes the Symbol, PubChem CID number, Expression Log Ratio, Expression P-Value, and molecular Type.

Symbol	PubChem CID	Expr. Log_Ratio	Expr. P_value	Type(s)
(2E,4E)-2,4-hexadienoic acid	643460	1,100	3,91E-02	chemical - endogenous mammalian
(R)-3-hydroxybutyric acid	92135	2,577	1,38E-02	chemical - endogenous mammalian
(S)-2,4-diaminobutanoic acid	134490	-1,284	1,30E-02	chemical - endogenous non-mammalian
(S)-salsolinol	91588	-2,110	4,83E-03	chemical - endogenous mammalian
1-11-undecanedioic acid	15816	-1,880	2,64E-03	chemical - endogenous mammalian
1-myristoyl-lysophosphatidic acid	9547180	1,322	1,25E-05	chemical - endogenous mammalian
10,16-dihydroxyhexadecanoic acid	441449	-2,426	1,92E-03	chemical - endogenous non-mammalian
11-hydroxyandrosterone	10286365	1,377	1,09E-02	chemical - endogenous mammalian
14-hydroxy-docosahexaenoic acid	11566378	1,125	1,21E-02	chemical - endogenous mammalian
1beta-hydroxycholic acid	5283893	-1,842	6,47E-03	chemical - endogenous mammalian
2-hydroxycaproic acid	99824	-1,335	1,34E-02	chemical - endogenous mammalian
3-carboxy-4-methyl-5-propyl-2-furanpropanoic acid	123979	-1,507	3,68E-03	chemical - endogenous mammalian
3-dehydrosphinganine	439853	2,141	4,74E-02	chemical - endogenous mammalian
3-hydroxydodecanedioic acid	16663321	-2,835	2,51E-03	chemical - endogenous mammalian
3-hydroxytetradecanedioic acid	20848956	-4,754	2,03E-03	chemical - endogenous mammalian
4,4-dimethyl-5alpha-cholesta-8,14,24-trien-3beta-ol	443212	1,005	5,87E-03	chemical - endogenous mammalian
4-cresol sulfate	4615423	1,098	4,70E-02	chemical - endogenous mammalian
6-isopentenyladenosine	24405	2,720	1,21E-02	chemical - endogenous non-mammalian
adenine	190	1,135	1,19E-04	chemical - endogenous mammalian
ascorbic acid	54670067	-4,726	1,02E-03	chemical - endogenous mammalian
ascorbyl palmitate	54680660	-2,595	2,05E-03	chemical drug
capryloylglycine	84290	-2,519	8,57E-04	chemical - endogenous mammalian
cis-4-octenedioic acid	11805205	-1,263	4,73E-04	chemical - endogenous mammalian

cis-aconitic acid	643757	-2,029	7,33E-04	chemical - endogenous mammalian
cyclohexanecarboxylic acid	7413	-1,442	5,92E-03	chemical reagent
D-alanyl-D-alanine	5460362	-1,113	3,88E-02	chemical - endogenous mammalian
delta-valerolactone	10953	-1,161	3,67E-03	chemical - endogenous non-mammalian
deoxyuridine	13712	1,426	4,76E-02	chemical - endogenous mammalian
dihydroxyphenylethylene glycol	91528	-1,085	6,68E-03	chemical - endogenous mammalian
dioctyl sulfosuccinate	11339	-2,281	1,42E-02	chemical drug
docosanamide	76468	-2,363	1,04E-02	chemical - endogenous mammalian
dodecanedioic acid	12736	-3,008	2,49E-03	chemical - endogenous mammalian
eicosapentaenoylethanolamide	5283450	2,020	1,50E-03	chemical - endogenous mammalian
erythro-3-hydroxy-L-aspartic acid	14463	-1,734	4,23E-02	chemical - endogenous non-mammalian
ethanolamine	700	1,400	4,05E-03	chemical - endogenous mammalian
ethyl oleate	5363269	1,828	1,27E-03	chemical - endogenous non-mammalian
glutathione	124886	4,506	2,99E-02	chemical - endogenous mammalian
glutaurine	68759	-1,131	2,82E-02	chemical - endogenous mammalian
GMP	135398631	-1,068	1,19E-02	chemical - endogenous mammalian
guanine	135398634	1,017	9,54E-03	chemical - endogenous mammalian
heneicosanoic acid	16898	-1,261	4,82E-03	chemical - endogenous mammalian
heptanoic acid	8094	-1,878	3,79E-03	chemical - endogenous mammalian
IMP	135398640	-1,356	2,34E-02	chemical - endogenous mammalian
N-acetyl-L-glutamic acid gamma-semialdehyde	192878	-1,027	3,84E-02	chemical - endogenous mammalian
N-arachidonoylalanine	40846579	1,508	2,16E-03	chemical reagent
N-arachidonylglycine	5283389	1,744	7,08E-03	chemical - endogenous mammalian
nonadecanoic acid	12591	-2,151	3,17E-05	chemical - endogenous mammalian
norethindrone	6230	1,075	1,14E-02	chemical drug
oleic acid	445639	2,092	3,79E-02	chemical - endogenous mammalian
pantetheine	439322	2,332	9,34E-03	chemical - endogenous mammalian
pimelic acid	385	-2,105	2,24E-03	chemical reagent
pyridoxal	1050	1,288	4,00E-02	chemical - endogenous mammalian

S-glutathionyl-L-cysteine	10455148	-1,133	1,28E-02	chemical - endogenous mammalian
saccharin	5143	-2,777	1,46E-03	chemical reagent
sebacic acid	5192	-2,247	1,94E-03	chemical - endogenous mammalian
shikimic acid	8742	-1,970	1,98E-03	chemical - endogenous non-mammalian
stipitatic acid	20501	-1,809	2,07E-03	chemical - endogenous non-mammalian
testosterone	6013	1,124	1,05E-02	chemical - endogenous mammalian
thymidine	5789	2,441	2,24E-02	chemical - endogenous mammalian
thymine	1135	1,492	2,95E-03	chemical - endogenous mammalian
toluene-cis-1,2-dihydrodiol	440399	-1,818	2,95E-03	chemical - endogenous non-mammalian
trans,trans-2,4-hexadienal	637564	-1,432	2,96E-03	chemical toxicant
traumatin	5312889	-1,937	1,95E-03	chemical - endogenous non-mammalian
